# A Collection of Memoirs on Military Medicine, &c.

**Published:** 1846-01

**Authors:** 


Recueil de Memoires de Medicine, de Chirurgie et de Phamacie Militaires. Vol. 58. 8vo. pp. 396. Paris, 1845.
A Collection of Memoirs on Military Medicine, &c.

Although their African possessions have otherwise proved of little service to the French nation, they have afforded a wide and useful field of research for the army medical officers, who have availed themselves of it in the most diligent and praiseworthy manner. We well know in this country the value of a corps of medical observers of this kind, not a few of the improvements in our modes of treating diseases having originated with our naval, military, and Indian practitioners. The medical officers attached to the army of Algeria are likewise, from time to time, transmitting to their mother country valuable memoirs and observations, which are submitted to the Army Council of Health, and published biennially in the "Recueil," whose latest volume we have now in hand. We observe in the practice of the African medical officers an emancipation from the shackles of hypothesis, and an energy and appropriateness of treatment—the result of an independent spirit of observation,—which sooner or later must exert a beneficial influence on the more theoretical and timid practitioners at home. The present volume is chiefly devoted to the consideration of the important subject,

### The Diseases of the Liver in Algeria.

Although the practitioners of this country have been made, through the writings of their brethren in India, familiar with the influence of hot climates in generating and modifying diseases of the liver, it can but be interesting and useful to note the conclusions other inquirers have arrived at under analogous circumstances. These will be found confirmatory of those we are already in possession of.

The first paper, upon *Hyperæmia of the Liver,* is from the pen of Dr. Haspel. This officer, who seems a very intelligent practitioner, contributed an essay to a former volume of the "*Recueil,*" upon *Abscess of the Liver,* to which we shall have occasion to advert presently. In it he observed that that disease seemed frequently to present itself in a sudden and insidious manner, by reason of the practitioner not having had the opportunity, or not having availed himself of it, of observing the antecedent condition of the organ. In the present paper he endeavours to indicate a condition of the liver, which in one of its varieties frequently terminates in the formation of abscess. He terms it *Hyperæmia,* and believes that it is too frequently overlooked as trifling, in consequence of attention not being paid to the evils it may eventually give rise to. The *Hyperæmia* may present itself to notice either in an *active,* or in a *passive* form, the former being distinguishable from hepatitis only by its greater mildness.

*Active Hyperæmia of the Liver.*—In the hot months of June, July, and

August, numbers of soldiers apply to the hospital, having a deranged condition of the digestive organs with predominance of symptoms referrible to the liver, as loaded tongue, constipated bowels, icteric conjunctivæ, pain in hypochondrium, &c. Sometimes there is only anorexia or difficult digestion complained of, and pain or tenderness in the region of the liver is only detected by a practitioner alive to the true nature of the case. The patient is usually relieved, but relapse is exceedingly liable to occur, and the disease then takes on a chronic form, the patient often temporarily improving so as to lead to the belief that his disease is far milder than it really is. Persistence in the loss of appetite and an anormal condition of the bowels, even when there is little or no pain, serve to indicate the chronic form. Could the soldier be placed now under favourable hygienic circumstances as regards diet, fatigue, exposure, &c. he might recover; but as the very contrary is the case, he too often, after the cold weather has commenced, returns to the hospital, which he had quitted in good spirits, having a yellow and squalid countenance, and a considerable tumefaction in the right hypochondrium. When these patients die, the liver is found much engorged, sometimes softened and sometimes denser in structure, usually of a reddish colour, but diversified by other shades. When they have been cut off at an earlier stage by some complication, the liver may be healthy in appearance, but is usually voluminous, and always the seat of more or less local or general sanguineous congestion.

*Passive or Hypostatic Hyperæmia.*—Persons of weak constitutions suffer severely in Africa when the prolonged and burning heats of summer are exchanged for the cold, wet, and variable temperature of autumn—especially if from any cause they may have been the subjects of chronic hyperæmia of the liver. The fluids repelled from the surface become accumulated in the internal viscera, especially the liver—producing a passive hyperæmia, which, however, may easily pass into a state of active inflammation, and give rise to abscess or ramolissement. There is here, too, more or less derangement of the digestive organs, uneasiness or pain and tumefaction of the hypochondrium, and icteric tinge, especially of the conjunctivæ. In some cases this last, with colourless stools, and deep-coloured urine, are the only symptoms.

The *chronic form* of passive hyperæmia (the *hepatitis occulta sive typhoida* of the ancients), is however far more dangerous than this, as, from its insidious approach, it frequently commits irreparable destruction before even its existence is discovered. So little, sometimes, is the disturbance produced in the economy, that a person having an enormous liver may yet retain all the appearances of good health, and patients may die without suffering any pain whatever in the hypochondriac or epigastric regions. In other cases, the straw-coloured complexion, peculiar physiognomy, and great wasting, plainly indicate the implication of some vital organ. In robust subjects, there is more re-action, in the shape of remittent and hectic fever. The liver is far more enlarged than in the acute form, the blood pouring out on incising the organ, and especially from its right lobe. More or less ramolissement exists, and vast purulent deposits are frequently found.

*Treatment.*—In the *active form* careful diet and mild aperients suffice in slight cases, and in severer ones, free bleeding and afterwards an emetic or two. In the chronic stage, moxas and blisters are useful; but a sea-voyage, return to Europe, and a course of mineral waters, are the best measures. In the *acute form* of *passive hyperæmia* general bleeding is rarely proper, as inertia of the secretory apparatus and atony of its circulation are the characteristics of this state. Purgatives and emetics are indicated, and mercurial frictions may afterwards be used over the enlarged liver. In the *chronic* form a supporting regimen, tonics, and stimuli are called for.

The author thus sums up the points of distinction between active and passive hyperæmia.

"*Active Hyperæmia.*—1. Is especially developed in the months of June, July and August. 2. Dry, arid, heat is one of its most active causes. 3. It attacks robust and bilious habits by preference. 4. It is often accompanied or preceded by a slight phlegmasia of the superior portion of the intestinal canal. 5. Its causes especially affect the super-diaphragmatic organs, producing parotiditis, deafness, epistaxis, &c. 6. It generally terminates by resolution, sometimes by hypertrophy of the organ, rarely by suppuration. 7. The lesions are found indifferently in any portion of the liver. 8. Local and general bleeding form the basis of treatment, purgatives are only sometimes useful, and tonics are contra-indicated.

"*Passive or Hypostatic Hyperæmia.*—1. Is observed especially in September, October, and November. 2. Common causes are damp and cold weather suddenly following great heats, and repeated paroxysms of intermittent fever. 3. It attacks weak subjects, whose viscera are of uncompact texture, and especially when these persons are debilitated by fatigue, chronic disease, diarrhœa, &c. 4. It most frequently co-exists with irritation of the large bowels, so that it is sometimes difficult to say which organ is first attacked. 5. The crises are operated upon the sub-diaphragmic organs, producing dysentery, hæmorrhoids, &c. 6. Suppuration is a common termination, hypertrophy a rare one. 7. The right lobe is especially affected. 8. Bleeding is rarely proper, mild purges are indicated in the acute, but rarely in the chronic form, when tonics are essential."

The next paper relates to an interesting subject, which we have alluded to in the reviews of Mr. Webb's Pathologica Indica and Dr. Budd's Treatise on Diseases of the Liver, contained in our last volume. It is entitled

### *On the Coincidence of Hepatitis and Abscess of the Liver, with the Endemic Diarrhœa and Dysentery of the Province of Oran. By Dr. Catteloup.*

Of 157 deaths that occurred during eighteen months in the hospital-practice of Dr. Catteloup, and in two-thirds of which diarrhœa or dysentery had prevailed during life, there were, in 65 cases, notable marks of disease of the liver, such as congestion, anormal colour, fatty degeneration, abscess (of which there were 20 examples), ramollissement, &c.

In analysing Dr. Catteloup's remarks, we may also allude to Dr. Haspel's Paper upon *Abscess of the Liver*, which appeared in a former volume of the "Recueil."

1. *Etiology.*—(1). *Climate.*—The prevalence of a form of hepatitis terminating in abscess seems peculiar almost to this province of Oran, for with the exception of two cases occurring at Algiers, none others have been reported from other parts of French Africa. MM. Catteloup and Haspel concur in attributing the production of the disease to the frequent and sudden vicissitudes of temperature rather than to intense heat. In fact, the agency of heat chiefly operates in Summer by congesting the liver, and rendering it liable to become inflamed under the influence of the chilling of the surface produced by the cold mornings and evenings of Autumn. In this part of Africa, there are no stagnant or marshy districts capable of generating intermittent-producing miasmata. The Arabs, however, who are so much exposed to the vicissitudes of the season, and take such inefficient precautions against them, are seldom the victims of the disease; but then a long training from infancy has blunted the susceptibility of the cutaneous surface, and these men avoid the stimulating diet of the Europeans. It seems that length of residence in Algeria increases the liability of the soldier to hepatic abscess, and in none of the 20 cases cited had the patient been less than a year in the colony. Dr. Catteloup took some pains to discover whether soldiers brought from the northern parts of France, or those who came from districts whose temperature more nearly resembled that of Algeria, were most liable to the disease. He found that, as regards simple dysentery, there was little difference in this respect, but that this became complicated with hepatitis and abscess most frequently in soldiers who had been drawn from the north of France.

(2). *Habits of Life.*—Unless the European upon arriving in a warm climate adopts the temperate habits of its inhabitants he is likely to fall a victim to his own imprudence. The stimulating food and drink even requisite in cold climates are poisonous here. Almost all the cases recorded in this Essay occurred in persons of intemperate habits. The soldier, too, has to struggle against the *adverse hygienic conditions* in which he is placed; and truly, according to the picture drawn by these and other writers, the condition of the French soldier in Algeria as regards food, exposure to weather, excessive duty, bad lodging, &c. is truly pitiable. The superior position of the officers in these respects, and their less dissipated habits, nearly exempt them from the disease we are treating of. M. C. attributes much mischief to the enormous quantity of ripe and unripe fruit consumed by the soldiers, especially when they are only partially convalescent from other diseases.

(3). *Constitution.*—Endemic disease especially affects the feeble, desponding, nostalgic, young, or lymphatic subject. In such, the viscera easily become congested, and we have diarrhœa or dysentery set up. Acute hepatitis terminating in suppuration usually occurs in the more robust subjects.

(4). *Age.*—From 21 to 36 are the ages during which the liver most frequently becomes diseased. In only two out of the 20 cases of abscess was the patient less than 25 years old.

2. *Pathological Anatomy.*—The following general results of post-mortem examinations in hepatitis may be stated. The *lungs* were usually found exsanguineous and very rarely tuberculous. The *heart* usually small and healthy. The *stomach* and *small intestines* were mostly found unaffected, although symptoms resembling gastritis or duodenitis may have prevailed. In the *large intestines*, however, every possible mark of inflammatory action, from a mere blush to perforating ulcers and gangrene, were observed. The cæcum and sigmoid flexure were the especial seats of disease; but however extensively the ulceration, &c., may have involved the intestine, there were always, at intervals, small healthy portions to be observed. This same almost constant coincidence of inflammation of the lower portion of the intestinal canal, with attendant diarrhœa or dysentery, (so contrasted with the affection of the upper portion of the canal, and constipation observed in Europe), was also observed by M. Haspel in his cases. As to the *liver* itself, the first degree of change in *general hepatitis* differs only from congestion, inasmuch as the blood, instead of running out on incision, seems identified with the granular structure of the liver. To this succeeds ramollissement, with different shades of colour, in which the texture of the organ is torn easily, although it does not lose its cohesiveness, like softened spleen. As matter is formed, greyish or whitish shades are produced by the deposition of the molecules of pus among the granulations of the organ, preparatory to constituting diffused suppuration, and eventually sometimes a vast abscess. Usually, however, the hepatitis and consequent abscess are more *partial*. If the abscesses are numerous, they are not usually voluminous, but in most cases there is but one. The right lobe is by far the most commonly affected, and the abscess is much oftener deep-seated than superficial. The process of formation of these isolated abscesses, from the period of congestion and discoloration of portions of the liver to the generation of a false membrane and their subsequent enlargement, is well described, but we have not space to do more than recommend the perusal. There is, however, one change, frequently found in the liver of dysenteric and other patients in Algeria, which, from its great interest, we must allude to;—we mean the *fatty degeneration*. M. Catteloup observes,—

"In the course of our researches we have remarked the frequency of a change of structure in subjects, who, offering every appearance of phthisis, yet were quite exempt from tubercle—I mean the yellow-looking and fatty liver. Every one is aware of the rarity of tubercles in Africa. Can it be that they are replaced by a morbid condition of the liver whose frequency is almost as great relatively to other affections, as it that of pulmonary tubercles compared to other diseases in France. Do the modifiers of the animal economy in Algeria possess the power of destroying in the *thoracic* lung the disposition to contract this pathological condition, but at the expense of its production in the *abdominal* lung? In France, the coincidence of the fatty liver and phthisis is frequently observed: in Algeria, this morbid condition is observed alone, no disease existing in the lung."

The rarity of the prevalence of pulmonary phthisis in Algeria may be judged of by the fact, that M. Haspel, in 1841, only found three persons affected with it out of 1480 hospital patients; and only one of 138 deaths was caused by it. So, in 1842, in all the hospital establishments of Algeria, of 8485 patients there were but 13 phthisical; and but 10 out of 871 deaths occurred from phthisis!

3. *Symptoms.*—Practitioners would be often disappointed if they always

F *

expected to see these as distinctly marked as they are enumerated in books. This is only so when the cases are seen early, and pursue an active course. In others, large abscesses and other ravages may have taken place with *little or no indication of their existence,* unless these had been specially sought for. The concurrent affection of the intestine sometimes furnishes nearly the only symptom, and masks those of the other disease. M. Haspel delivers many cautionary observations as to the insidious manner in which the disease may come on, among which are the following.

" This affection is liable to take on the appearance and symptoms of most of the chronic affections of the chest and alimentary canal, or it may become concealed by the symptoms of other diseases existing simultaneously with it, as diarrhœa or dysentery. Sometimes it offers all the characters of an apyretic gastro-intestinal affection, and at others is accompanied by an intermittent fever of the tertian type. The degree of pain it gives rise to is very different, and sometimes this does not exist at all. Many patients only complain of pain when they walk or employ other exertion. The pain may be pungent, lancinating, and acute, like that of pleuritis, or, at other times, dull and obtuse, extending from the liver to the lungs, and rising sometimes as high as the shoulder."

M. Catteloup presents a general sketch of the symptoms in the three separate stages of irritation, inflammation, and suppuration. But, in place of transcribing this, we prefer following him in his detail of the separate signs of the disease.

(1). *Pain.*—This is the most important symptom, and one which, even alone, is sometimes sufficient to indicate the impending danger to the liver. It must, however, not be confounded with that affecting the large intestine in dysentery. It may be distinguished from hepatalgia by the more severe, lancinating, and paroxysmal character of the latter. It is not always confined to the right side, but may sometimes extend to the epigastrium, or even the left hypochondrium. At others, it is absent entirely. The pain in the right shoulder, set down in some works as so constant a sign, is seldom met with in Algeria.

(2). *Tumefaction.*—When the size of the liver is augmented, it can hardly be overlooked if percussion is employed ; but sometimes the organ maintains its normal size, especially if the abscess be small or deep-seated, so that little information is usually obtained from this sign in partial hepatitis. Moreover, after a long residence in Africa, the liver, although otherwise healthy, acquires a larger size than in France. However, in hepatitis, the organ is oftener found enlarged than of normal size, and never less than natural.

(3). *Icterus* is only indicative of hepatitis when combined with the above signs, for it is equally present in hepatalgia and other diseases. M. Haspel states that he seldom found icterus present in the cases of hepatic abscess he reports as occurring at Oran ; but M. Simon found it very marked in others occurring at Mascara in the same province. But the patients at Oran were old cases and debilitated subjects, and those at Mascara were men in robust health, who were suddenly attacked while performing forced marches under a burning sun.

(4). *Pulse and Febrile Re-action.*—During the stage of irritation the pulse may be quiet and normal; but when inflammation is set up, it becomes quick and hard. If such change takes place in the pulse during the progress of a dysentery, we have every reason to believe that hepatitis has complicated the disease. M. Haspel represents the pulse as continuing small and normal, or even slower than natural, until towards the termination of the disease, when it becomes rapid, and increases in quickness and energy *pari passu* with the prostration of the patient. An increase of number and power of the pulse, during the night, especially if accompanied by sweating, should give rise to a very unfavourable prognosis, even when there is no apparent sign of local disorder. A paroxysmal fever may precede the congestion and inflammation of the liver, but in this part of Africa it seldom declares itself until the disease is fully-formed. The shivering announcing the formation of matter has not unfrequently been mistaken for intermitting fever until quinine has been exhibited unsuccessfully. The *respiration* is more or less embarrassed in proportion to the suddenness of the encroachment of the enlarged liver upon the thoracic cavity. To whatever extent the patient's suffering may eventually be carried, or his prostration extend, no re-action takes place in the brain, the intellectual powers continuing clear and complete to the last.

4. Although the *Prognosis* is of the gravest character in hepatic abscess, as most of the patients die, yet recovery occasionally takes place after the evacuation or the absorption of their contents. Cicatrices of former superficial abscesses have been several times met with. A curious case is related in a former number of the "Recueil." A soldier, after five years' service in Algeria, was admitted with hepatic abscess into one of the hospitals there in 1837. It discharged itself spontaneously under the right false ribs, and he resumed his duties. He, however, since has repaired to the hospital, at intervals of about three months, in order to have the abscess punctured, and issue given to new collections of matter; so that, at the period of the report, in 1840, such punctures had been made 24 times.

Before proceeding to the consideration of the treatment of this compound disease, hepatitis and dysentery, M. Catteloup alludes to the difference of opinion which prevails as to the determination of which of its two elements should be placed in the position of cause, and which in that of effect. He observes he has never seen a case in which the hepatitis should be looked upon as other than the effect, diarrhœa or dysentery having in all preceded the hepatic symptoms. He agrees in the *modus operandi* by which this is brought about, as stated by M. Bouillaud, and also indeed by Dr. Budd (see p. 500 of our last No.), viz. the absorption by the venous radicles of some of the diseased matters abounding on the disorganized intestinal surface, and their consequent conveyance into the portal system. The liver, already predisposed by the congested state produced by the heats of Summer, or an injudicious regimen, then becomes irritated and inflamed.

"But this action is confined to the hepatic organ. The purulent generation is not here, as in phlebitis, a general phenomenon localizing itself in all the viscera by the transport of a puriform inflammation. In the description of cases we are engaged with, we have never seen any trace of venous inflammation, or any appearance of pus circulating in the blood. It is not then a true purulent resorption, whose symptoms are usually so formidable, but merely the transport of a deleterious substance, of a certain morbid product by the blood, which, however inappreciable by our means of analysis, is no less real."

*Treatment.*—The treatment of this compound disease is often difficult, for vigorous procedures appropriate for the hepatitis, are by no means so for the dysentery. In the early stage of irritation, when bilious diarrhœa is the principal symptom, the author chiefly relies upon the due regulation of diet and other hygienic precautions. He forbids the endeavour to check the diarrhœa by means of emetics, the effect of which would only be, if successful, to congest the liver. If we wish to prevent suppuration, the inflammatory stage must be met by a full general bleeding, and that even where mere local tenderness is our only indication of treatment. Leeches or cupping must follow, and in cases where the dysenteric symptoms are severe, and the patients powers low, we must content ourselves with these latter, or even with powerful revulsives, as moxas, cauteries, &c. Mere dysentery, without fever, or great pain, is best treated by careful diet and starch glysters, and where there is pain, by leeching or cupping over the colon. If the pain is great, and seated near the liver, and the patient plethoric, general bleeding, and afterwards numerous relays of leeches, must be had recourse to, applying the latter especially to the iliac fossæ. Emetics are to be discarded as often useless for the dysentery, and injurious to the hepatitis. As the active symptoms and fever diminish, opiates become very useful, and may be advantageously combined with calomel and ipecacuanha.

There is always great danger of a relapse, especially if the diet is too full after the first relief of urgent symptoms. In relieving the dysentery by medicines alone, the importance of attention to hygiene has been too much overlooked, which accounts for a great deal of the frightful mortality once so prevalent in the barracks in Algeria, and now so much diminished. Above all things, the patients should not be crowded together. Two dysenteric patients placed in proximity soon become adynamic, and diarrhœa from a like cause quickly passes into dysentery.

During the suppurative stage, antiphlogistics must be laid aside, and means for maintaining the general strength resorted to—still employing external emollient and counter-irritant applications ; and administering internally, opiates, Segond's pills, quinine, &c. Both M. Catteloup and M. Haspel believe the cases are very rare where opening the abscess by incision or caustic would be justifiable, or even possible.

We regret our space prevents our transcribing any of the twenty cases detailed and very ably commented upon by M. Catteloup : but they, together with M. Haspel's cases in the 55th Vol. of the "Recueil," form a valuable body of information on the history of suppurative inflammation of the liver.

We hope this interesting subject will be pursued in a future volume of the "Receuil." The present position of the French in Algeria indicates there will be no lack of *materiel* for some time to come.

